# Incidence of Pediatric Urinary Tract Infections Before and During the COVID-19 Pandemic

**DOI:** 10.1001/jamanetworkopen.2023.50061

**Published:** 2024-01-03

**Authors:** Danni Liang, Marie E. Wang, Alex Dahlen, Yungting Liao, Andrew C. Saunders, Eric R. Coon, Alan R. Schroeder

**Affiliations:** 1 Department of Pediatrics, Stanford University School of Medicine, Palo Alto, California; 2 Quantitative Sciences Unit, Department of Medicine, Stanford University School of Medicine, Palo Alto, California; 3 Department of Pediatrics, Primary Children’s Hospital and University of Utah School of Medicine, Salt Lake City

## Abstract

**IMPORTANCE:**

Urinary tract infection (UTI) is common in children, but the population incidence is largely unknown. Controversy surrounds the optimal diagnostic criteria and how to balance the risks of undertreatment and overtreatment. Changes in health care use during the COVID-19 pandemic created a natural experiment to examine health care use and UTI diagnosis and outcomes.

**OBJECTIVES:**

To examine the population incidence of UTI in children and assess the changes of the COVID-19 pandemic regarding UTI diagnoses and measures of UTI severity.

**DESIGN, SETTING, AND PARTICIPANTS:**

This retrospective observational cohort study used US commercial claims data from privately insured patients aged 0 to 17 years from January 1, 2016, to December 31, 2021.

**EXPOSURE:**

Time periods included prepandemic (January 1, 2016, to February 29, 2020), early pandemic (April 1 to June 30, 2020), and midpandemic (July 1, 2020, to December 31, 2021).

**MAIN OUTCOMES AND MEASURES:**

The primary outcome was the incidence of UTI, defined as having a UTI diagnosis code with an accompanying antibiotic prescription. Balancing measures included measures of UTI severity, including hospitalizations and intensive care unit admissions. Trends were evaluated using an interrupted time-series analysis.

**RESULTS:**

The cohort included 13 221 117 enrollees aged 0 to 17 years, with males representing 6 744 250 (51.0%) of the population. The mean incidence of UTI diagnoses was 1.300 (95% CI, 1.296-1.304) UTIs per 100 patient-years. The UTI incidence was 0.86 per 100 patient-years at age 0 to 1 year, 1.58 per 100 patient-years at 2 to 5 years, 1.24 per 100 patient-years at 6 to 11 years, and 1.37 per 100 patient-years at 12 to 17 years, and was higher in females vs males (2.48 [95% CI, 2.46-2.50] vs 0.180 [95% CI, 0.178-0.182] per 100 patient-years). Compared with prepandemic trends, UTIs decreased in the early pandemic: −33.1% (95% CI, −39.4% to −26.1%) for all children and −52.1% (95% CI, −62.1% to −39.5%) in a subgroup of infants aged 60 days or younger. However, all measures of UTI severity decreased or were not significantly different. The UTI incidence returned to near prepandemic rates (−4.3%; 95% CI, −32.0% to 34.6% for all children) after the first 3 months of the pandemic.

**CONCLUSIONS AND RELEVANCE:**

In this cohort study, UTI diagnosis decreased during the early pandemic period without an increase in measures of disease severity, suggesting that reduced overdiagnosis and/or reduced misdiagnosis may be an explanatory factor.

## Key Points

**Question:**

Did the incidence of pediatric urinary tract infection (UTI) diagnoses and outcomes change during the COVID-19 pandemic?

**Findings:**

In this cohort study of 13 million children with private insurance, the incidence of UTI was 1.30 cases per 100 patient-years, with notable variation by age, sex, and circumcision status. Compared with prepandemic trends, UTI diagnoses decreased by 33% during the early pandemic without associated changes in disease severity.

**Meaning:**

This investigation provides updated data on UTI incidence in children; while the mechanism for decreased UTI incidence during the pandemic is unknown, a decrease in misdiagnosis and overdiagnosis may play a role.

## Teaser

This cohort study examines the incidence of urinary tract infection diagnosed in children during the COVID-19 pandemic, with comparison of prepandemic, early pandemic and midpandemic rates.

## Introduction

Urinary tract infections (UTIs) are one of the most common bacterial infections in children^1^ and the leading cause of bacterial infections in infants,^2,3,4^ yet the population incidence for all children is still largely unknown. Urinary tract infections are typically treated with antibiotics and may lead to hospitalization, genitourinary imaging, follow-up visits, and surgical intervention.^1,5^ Controversy remains surrounding the optimal UTI diagnostic criteria, the need for prompt diagnosis, and the role of imaging and prophylactic antibiotic therapy.^6,7,8,9,10^

The COVID-19 pandemic was associated with substantial decreases in health care use and viral respiratory infection transmission from social distancing measures.^11,12,13,14^ These shifts offer a natural experiment to gain insight into testing, treatment, and outcomes for UTI. For a nontransmissible infection such as UTI, decreased diagnosis during the pandemic would likely be primarily associated with decreased pursuit of medical care and/or a reduction in the evaluation of fever as a result of the near disappearance of most circulating viruses. Fewer UTI diagnoses during the pandemic in the face of unchanged severe outcomes may suggest that overdiagnosis and/or overtreatment were previously occurring.

While many investigations have described changes in patterns of medical care use and trends in common childhood diagnoses during the pandemic,^15,16,17,18,19,20,21,22,23^ studies have not looked specifically at the incidence of UTI diagnoses in both ambulatory and inpatient settings across community and children’s hospitals. The aims of this study were to provide estimates of population-level UTI incidence and assess changes in UTI diagnoses and measures of UTI severity, such as hospitalizations and intensive care unit (ICU) admissions, during the COVID-19 pandemic.

## Methods

### Study Design

We conducted a retrospective observational cohort study from 2016 to 2021, using US commercial claims data from the Merative MarketScan Research Database (MarketScan), which captures data on approximately 5 million privately insured enrollees annually younger than 18 years.^24^ This national database includes patient demographic characteristics (age in years, sex, and region), pharmaceutical claims, procedural claims, and inpatient and outpatient diagnosis codes.^25^ Race and ethnicity information was not available in the MarketScan Research Database. The Stanford University Institutional Review Board approved the use of this deidentified database for this study. This report follows the Strengthening the Reporting of Observational Studies in Epidemiology (STROBE) reporting guideline for observational studies.^26^

### Study Population

The full cohort consisted of any patient aged 0 through 17 years with at least 1 day of coverage in MarketScan with a drug plan. Patients enter the database at coverage start and are no longer a part of the cohort at either coverage end or age 18 years or older. Age categories were subclassified into 4 different groups based on risk factors such as bowel and bladder habits, school attendance, and puberty and sexual activity: ages 0 to 1, 2 to 4, 5 to 11, and 12 to 17 years.

We opted a priori to examine a subgroup of infants aged 60 days or younger since these younger infants represent a unique population who are frequently hospitalized for evaluation of any potential infection.^27^ Although date of birth is not provided in the database, we used the first day of the birth hospitalization to estimate birth date^28,29^ and required 60 subsequent days of continuous insurance coverage with a drug plan. Circumcision status for these infants was determined by a *Current Procedural Terminology* code (eTable 1 in Supplement 1) for circumcision within 3 weeks of the birth hospitalization.

### Time Periods

To determine changes in UTI incidence during the pandemic, we compared the prepandemic period (January 1, 2016, through February 29, 2020) with the COVID-19 pandemic period (April 1, 2020, through December 31, 2021). The 2016 start date was selected as the beginning of the prepandemic period to allow for an adequate number of years to detect secular trends and avoid any potential classification issues from the *International Classification of Diseases, 9th Revision* to the *International Statistical Classification of Diseases and Related Health Problems, 10th Revision* (*ICD-10*) conversion in 2015. April 2020 was selected as the beginning of the pandemic period because there was variability in March regarding strict lockdown procedures. The pandemic period was further divided into the early pandemic (April 1 to June 30, 2020) when lockdown procedures in the US were more strict and viral respiratory infection volumes continued to be substantially lower than prepandemic volumes,^30^ and midpandemic (July 1, 2020, to December 31, 2021) when there was more variation in stay-at-home measures. These periods were also chosen based on previous investigations into overall pediatric health care use, which showed a large decrease during the first 3 months and then a subsequent partial recovery for many types of care.^11,18^

### Primary Outcome

Our primary outcome was the incidence of UTI diagnosis in any ambulatory or inpatient setting. Due to ongoing controversy surrounding the appropriate diagnostic criteria for UTI and because claims databases do not provide urinalysis or urine culture results, a UTI diagnosis code plus antibiotic treatment was used to define UTI diagnosis (hereafter referred to as UTI). Urinary tract infection diagnosis codes included all *ICD-10* codes corresponding to pediatric UTI, cystitis, and/or pyelonephritis (eTable 1 in Supplement 1). Previous work has reported that the positive predictive value of *ICD-9* codes for UTI in hospitalized patients is low (50%-61% using laboratory-confirmed UTI as the reference standard); however, the positive predictive value increases considerably (85%-93%) using provider-confirmed UTI via medical record review as the highest standard.^31^ For this reason, we opted to add the requirement of evidence of an accompanying claim for an antibiotic prescription fill with a course duration of 14 days or less within 7 days of the initial diagnosis or an inpatient antibiotic code (eTable 1 in Supplement 1) for UTI-related antibiotics (eTable 2 in Supplement 1).^31,32,33,34^ Any diagnosis of UTI occurring within 2 weeks of a previous diagnosis counted as a singular diagnosis due to potential return visits for the same infection.^32^ A 2-week time period was selected since the longest recommended duration for antibiotic treatment is 14 days. For infants aged 60 days or younger, we excluded diagnoses of UTI that occurred within 0 to 3 days of age due to an extremely low likelihood of UTI in this age group^35^ and the possibility that a maternal UTI diagnosis code could carry over into the birth hospitalization. In MarketScan, the principal diagnosis code is not necessarily the one listed in the primary diagnostic position of inpatient visits,^25^ and since outpatient billing codes do not have principal diagnosis codes,^36^ we allowed for UTI diagnosis codes to be in any position.

### Balancing Measures

We evaluated several secondary outcomes related to UTI severity. Our primary measure of UTI severity for the full pediatric cohort was hospitalization with UTI, which was defined as a hospitalization with a new diagnosis code for UTI and an admission date within 5 days of diagnosis. Our primary measure of UTI severity for the subgroup of infants aged 60 days or younger was ICU admission. Usually, any type of hospitalization would suggest increased severity of disease, but infants aged 60 days or younger are often routinely hospitalized for evaluation of any potential infection.^27^ In this population, hospitalization without ICU admission does not reflect severity of disease in younger infants as it would for older children. Therefore, we opted to use ICU hospitalization with UTI as our primary measure of UTI severity for infants aged 60 days or younger. Additional measures of UTI severity included dehydration, shock, sepsis, acute kidney injury, and length of stay 4 days or more. Measures of severity were defined using a combination of *ICD-10*, *Current Procedural Terminology*, and Healthcare Common Procedure Coding System codes to capture diagnoses (eg, shock and sepsis) and/or procedures (ie, intravenous fluid use for dehydration) that may be associated with certain diagnoses (eTable 1 in Supplement 1).

### Statistical Analysis

We report UTI as a population incidence of diagnoses per 100 patient-years, obtained by dividing the number of patients with the outcome (ie, total number of UTI diagnoses) in 1 month by the number of patient-years of MarketScan coverage each month. We calculated UTI incidence using Poisson 95% CIs. We used an interrupted time-series approach to evaluate changes possibly attributable to the pandemic while accounting for preexisting trends in UTI diagnosis and management. The output of our interrupted time-series model is a percentage change in incidence compared with a counterfactual if the pandemic had not occurred; each model results in an estimate for this percentage change and its corresponding 95% CI during both time periods, and we ran a separate model for each outcome. We calculated trends in UTI incidence by age group, sex, and circumcision status. Detailed methods of the statistical model are described in the eMethods in Supplement 1. All analyses were conducted in Python, version 3.8.5 (Python Software Foundation). Regression parameters were estimated using the statsmodels package, version 0.12.0. All tests were 2-sided and performed with a significance threshold of .05.

## Results

### Study Population

From 2016 to 2021, a total of 13 221 117 child enrollees were included in the MarketScan Research Database. Table 1 displays the distribution of patient demographic characteristic percentages at the cohort start since there were variations in exact percentages of enrollees in age group and region over time. The average patient contributed 1.5 (IQR, 0.9-3.1) years of continuous medical data in the cohort. There was near equal representation of males and females (6 744 250 [51.0%] vs 6 476 867 [49.0%]).

**Table 1.** Demographic Information for All Pediatric Enrollees

Characteristic	No. (%)	
	All children (age, 0-17 y)	Infant subgroup (age, ≤60 d)
Enrollees	13 221 117	572 367
Days of coverage, median (IQR)	549 (304-1098)	60 (60-60)
Sex		
Male	6 744 250 (51.0)	294 552 (51.5)
Female	6 476 867 (49.0)	277 815 (48.5)
Age at cohort start		
≤60 d	NA	572 367 (100)
0-1 y	2 445 521 (18.5)	NA
2-4 y	1 887 036 (14.3)	NA
5-11 y	4 726 153 (35.7)	NA
12-17 y	4 162 407 (31.5)	NA
Region at cohort start		
Northeast	1 991 344 (15.1)	103 151 (18.0)
North Central	2 780 704 (21.0)	120 373 (21.0)
South	5 827 551 (44.1)	259 610 (45.4)
West	2 545 599 (19.3)	85 320 (14.9)
Unknown	75 919 (0.6)	3913 (0.7)

Abbreviation: NA, not applicable.

### UTI Incidence

Overall, among the full cohort of children aged 0 to 17 years, the mean UTI incidence was 1.300 (95% CI, 1.296-1.304) UTIs per 100 patient-years. Urinary tract infection incidence per 100 patient-years by age was 0.86 (95% CI, 0.85-0.87) at age 0 to 1 year, 1.58 (95% CI, 1.57-1.59) at 2 to 5 years, 1.24 (95% CI, 1.23-1.25) at 6 to 11 years, and 1.37 (95% CI, 1.36-1.38) at 12 to 17 years. The incidence of UTI was substantially higher in females vs males (2.48 [95% CI, 2.46-2.50] vs 0.180 [95% CI, 0.178-0.182] per 100 patient-years).

For the subgroup of infants aged 60 days or younger, the incidence of UTIs was 0.87 (95% CI, 0.81-0.93) per 100 patient-years. Incidence was higher in uncircumcised male infants (1.42 [95% CI, 1.23-1.61] UTIs per 100 patient-years) compared with circumcised male infants (0.49 [95% CI, 0.40-0.58] UTIs per 100 patient-years) and female infants (0.94 [95% CI, 0.85-1.03] UTIs per 100 patient-years) (Table 2).

**Table 2.** Overall Incidence of UTI Diagnosis and UTI Hospitalizations, by Age and Sex

Variable	No. of children	UTI, No.	UTI per 100 person-years, (95% CI)	UTI with hospitalization, No. (%)	UTI with ICU hospitalization, No. (%)
All children (age, 0-17 y)					
Overall	13 221 117	349 583	1.300 (1.296-1.304)	7086 (2.0)	955 (<0.1)
Age, y					
0-1	2 445 521	20 503	0.86 (0.85-0.87)	2518 (12.3)	295 (1)
2-4	3 106 782	60 863	1.58 (1.57-1.59)	832 (1.4)	89 (<0.1)
5-11	6 062 925	127 089	1.24 (1.23-1.25)	1474 (1.2)	206 (<0.1)
12-17	5 782 973	141 128	1.37 (1.36-1.38)	2262 (1.6)	365 (<0.1)
Sex					
Female	6 476 867	325 259	2.48 (2.46-2.50)	5466 (1.7)	715 (<0.1)
Male	6 744 250	24 324	0.180 (0.178-0.182)	1620 (6.7)	240 (1)
Infant subgroup (age, ≤60 d)					
All infants	572 367	819	0.87 (0.81-0.93)	489 (59.7)	47 (6)
Female	277 815	429	0.94 (0.85-1.03)	240 (55.9)	24 (6)
Male, uncircumcised	100 640	235	1.42 (1.23-1.61)	153 (65.1)	16 (7)
Male, circumcised	193 912	155	0.49 (0.40-0.58)	96 (61.9)	7 (5)

Abbreviations: ICU, intensive care unit; UTI, urinary tract infection.

### Trends in UTI Incidence and Measures of Severity

Among the full pediatric cohort, there were 11.4% (95% CI, 6.5%-16.3%) fewer UTI diagnoses each year during the prepandemic period. During the early pandemic period, the incidence of UTI diagnosis decreased substantially (−33.1%; 95% CI, −39.4% to −26.1%) (Table 3) compared with a counterfactual if prepandemic linear and seasonal trends continued (Figure). Despite this decrease in UTI incidence during the early pandemic, hospitalizations with UTI for the full cohort decreased (−17.7%; 95% CI, −31.1% to −1.1%) and other measures of UTI severity did not show any statistically significant increases. Shock, sepsis, and length of stay decreased significantly during the early pandemic, while changes in dehydration and acute kidney injury were not statistically significant (Table 3). The most commonly coded measures of severity were dehydration (associated with 30.7% of all pediatric hospitalizations with UTI) and length of stay 4 days or longer (23.9%); the least common were shock (3.5%) and acute kidney injury (5.4%) (eTable 3 in Supplement 1). When stratified by age and sex, percentage change in UTI incidence during the pandemic did not significantly differ from trends in the overall pediatric cohort (Table 4).

**Table 3.** Percentage Differences for Outcomes During Early Pandemic and Midpandemic Periods Compared With Anticipated Prepandemic Trends^a^

Variable	Per 100 patient-years, % (95% Cl)			
	All children (age, 0-17 y)		Infant subgroup (age, ≤60 d)	
	Early pandemic, April-June 2020	Midpandemic, July 2020-December 2021	Early pandemic, April-June 2020	Midpandemic, July 2020-December 2021
UTI diagnoses	−33.1 (−39.4 to −26.1)^b^	−4.3 (−32.0 to 34.6)	−52.1 (−62.1 to −39.5)^b^	−9.8 (−25.5 to 9.3)
Hospitalizations with UTI	−17.7 (−31.5 to −1.1)^b^	10.3 (−45.1 to 121.4)	−73.4 (−86.3 to −48.5)^b^	3.8 (−12.8 to 23.6)
Measures of UTI severity				
ICU admission	0.0 (−23.4 to 30.7)	−37.4 (−63.7 to 7.9)	52.8 (−8.7 to 155.6)	−6.3 (−42.9 to 53.7)
Shock	−57.4 (−81.6 to −1.3)^b^	−0.1 (−5.5 to 5.6)	−1.8 (−4.4 to 0.8)	−45.4 (−81.7 to 62.5)
Sepsis	−50.8 (−62.9 to −34.9)^b^	11.6 (−37.4 to 98.8)	−50.0 (−72.6 to −8.8)^b^	−1.5 (−30.1 to 38.6)
Dehydration	−22.2 (−47.6 to 15.4)	65.9 (−19.7 to 242.5)	53.1 (−1.6 to 138.3)	19.8 (−5.3 to 51.6)
Acute kidney injury	1.8 (−22.2 to 33.2)	0.2 (−9.3 to 10.7)	17.4 (−0.4 to 38.5)	−19.0 (−49.4 to 29.8)
LOS ≥4 d	−34.4 (−50.9 to −12.3)^b^	1.2 (−47.7 to 96.0)	−59.8 (−75.3 to −34.7)^b^	−22.2 (−40.5 to 1.7)

Abbreviations: ICU, intensive care unit; LOS, length of stay; UTI, urinary tract infection.

^a^ Analysis performed using an interrupted time series.

^b^ Significant results.

**Figure.**
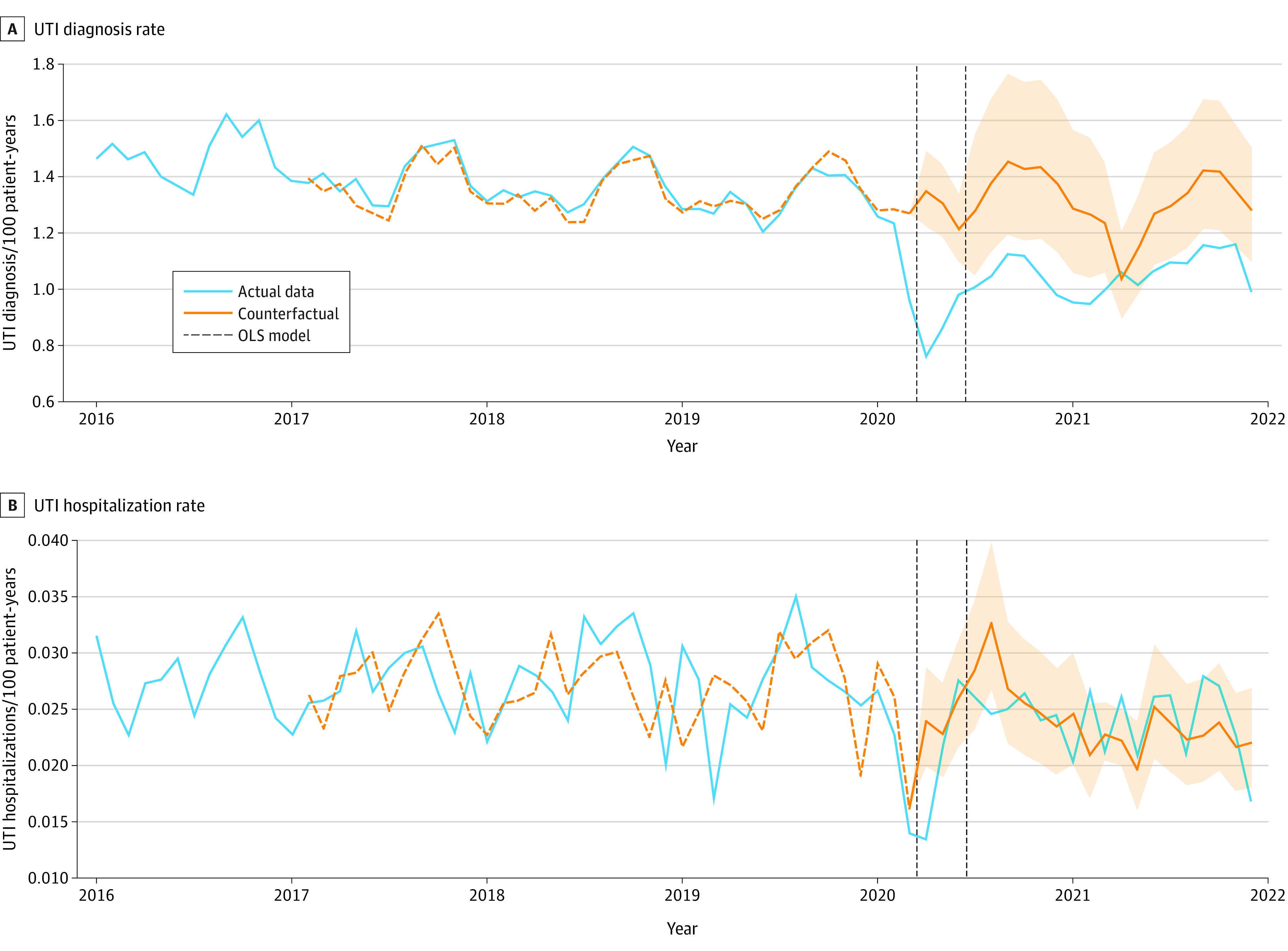
Interrupted Time Series of the Incidence of Urinary Tract Infection (UTI) Diagnoses and UTI Hospitalizations for Children Aged 0 to 17 Years From 2016-2021 The actual trend of data and a counterfactual where the pandemic did not occur and prepandemic trends had continued. Shaded area indicates 95% CI. The vertical black dashed lines represent the early pandemic period from April to June 2020. OLS indicates linear regression model.

**Table 4.** Percentage Change in UTI Diagnoses and UTI Hospitalizations Compared With Prepandemic Trends Stratified by Age and Sex in the Early Pandemic and Midpandemic Periods

Variable	UTIs per 100 person-years (95% Cl)		Hospitalizations per 100 person-years (95% Cl)	
	Early pandemic, April-June 2020	Midpandemic, July 2020-December 2021	Early pandemic, April-June 2020	Midpandemic, July 2020-December 2021
All children (age, 0-17 y)				
Age, y				
0-1	−10.1 (−17.9 to −1.5)^a^	−0.7 (−12.6 to 12.9)	−22.2 (−31.4 to −11.7)^a^	18.4 (−8.4 to 52.9)
2-4	−31.7 (−37.9 to −24.8)^a^	−7.9 (−25.8 to 14.4)	−2.5 (−40.6 to 59.8)	−11.1 (−38.7 to 28.9)
5-11	−38.6 (−45.6 to −30.7)^a^	−12.5 (−33.0 to 14.2)	−21.4 (−47.0 to 16.5)	−6.3 (−29.0 to 23.7)
12-17	−32.3 (−41.9 to −21.1)^a^	−9.8 (−21.8 to 4.2)	−14.7 (−33.0 to 8.8)	−3.6 (−27.3 to 27.6)
Sex				
Female	−33.4 (−39.7 to −26.4)^a^	−9.8 (−25.3 to 8.9)	−19.9 (−37.0 to 1.7)	1.4 (−16.3 to 22.7)
Male	−27.9 (−34.7 to −20.4)^a^	−10.2 (−27.7 to 11.5)	−8.7 (−23.2 to 8.4)	8.9 (−16.7 to 42.4)

Abbreviation: UTI, urinary tract infection.

^a^ Significant results.

All UTI diagnoses and measures of severity returned to near prepandemic rates after the first 3 months of the pandemic for the full pediatric cohort (−4.3%; 95% CI, −32.0% to 34.6%), and a seasonal variation was observed during the prepandemic period (Figure), when a consistent peak occurred annually in the fall to winter months. In the subgroup of infants aged 60 days or younger, the decrease in UTI incidence compared with prepandemic trends was even more pronounced. Both UTI diagnoses (−52.1%; 95% CI, −62.1% to −39.5%) and hospitalizations with UTI (−73.4%; 95% CI, −86.3% to −48.5%) (Table 3) decreased. Intensive care unit hospitalizations with UTI for infants aged 60 days or younger did not have a statistically significant change (52.8%; 95% CI, −8.7% to 155.6%) and other measures of severity for this subgroup demonstrated similar trends as the full pediatric cohort (Table 3).

## Discussion

In this retrospective cohort study of more than 13 million pediatric enrollees in a national commercial claims database, we provide updated information surrounding the population incidence and epidemiologic factors observed with UTI. We report substantial decreases in UTI diagnoses without a concurrent increase in measures of UTI severity during the early pandemic period compared with prepandemic trends. Our findings may provide insight into the association between diagnosis and outcomes for pediatric UTI.

Before this study, the overall population incidence of pediatric UTI was largely unknown. Many studies describe UTI prevalence,^4,37^ and any studies on incidence were conducted in multiple settings and mostly in groups with certain risk factors (eg, febrile infants, uncircumcised males, and adolescents), with variable reported incidence rates.^33,38,39,40,41,42^ In contrast, we describe the overall population incidence of pediatric UTI in children with private insurance from age 0 to 17 years with stratification by age, sex, and circumcision status. We observed a downward sloping of UTI incidence during the prepandemic period, with fewer UTI diagnoses each year before the pandemic. The reason for this trend is unclear and warrants further investigation. Potential explanatory factors include the adoption of stricter criteria for the diagnosis of UTI (eg, requirement for pyuria on the urinalysis) and diagnostic and antimicrobial stewardship efforts.^43^ This updated characterization of UTI epidemiologic factors in children may help inform clinical care, prioritization of resources and other policy discussions, quality improvement and stewardship efforts, and future research.

Several earlier investigations have noted either decreased or unchanged rates of UTI during the pandemic compared with prepandemic trends.^16,18,19,20,21,22^ However, these studies were limited to select settings of care (eg, outpatient only,^16^ inpatient pediatrics at only university-affiliated sites,^18,19^ US hospitals,^16,18,19^ and international hospitals^20,21,22^). Additionally, because these studies focused on numerous conditions, they did not use comprehensive lists of diagnosis codes, did not require evidence of antibiotic treatment for the definition of UTI, and did not examine severity measures.

Our finding that pediatric UTI incidence decreased substantially in the early pandemic without significant changes in measures of disease severity has several potential explanations. First, this trend could represent a reduction in the incidence of true UTI. However, given that UTIs are generally thought to be a nontransmissible infection, it is unclear how the pandemic and the associated lockdowns might have altered true UTI infection rates. Bowel and bladder habits may have changed, although the uniformity of decreased UTI incidence across all age groups, including younger infants, makes this explanation less likely.

Second, there is some evidence that viral respiratory infections apart from COVID-19 decreased substantially for several months, likely due, in part, to nonpharmaceutical interventions aimed at containing the pandemic.^12,13,14^ The pattern of circulation for viral infections may be associated with the diagnosis of UTI in at least 2 distinct ways. The observed decrease in UTI incidence in the early pandemic may be explained by decreases in UTI misdiagnosis (ie, false-positive findings, such as bacteriuria) and overdiagnosis (ie, true infections that resolve without treatment). In our study, we noted an interesting seasonal pattern to UTI diagnoses, where the peak incidence of UTI was seen in the fall months when some viral respiratory illnesses also predominate.^44^ The decrease in viral respiratory infections during the pandemic may have subsequently led to fewer evaluations for fever or other vague symptoms possibly attributable to UTI, which would decrease the potential for an incorrect diagnosis, or misdiagnosis, of UTI.^7^ More children presenting to pediatric care facilities in the fall, whether related to viruses or not, could explain the increase in UTI. However, viral respiratory infections and pediatric care visits tend to be at their highest during the winter, yet we saw a reduction in UTI during winter months compared with fall months. Therefore, it is not clear that the seasonal spikes we observed are attributable to increased health care use or fever evaluations due to viral respiratory infections.

Alternatively, if viral respiratory infections predispose children to UTIs, then reductions in these infections could also lead to a decrease in the incidence of true UTI. However, to our knowledge, there is no conclusive evidence that viral infections increase the risk of UTI. Although some studies have described an association between viral bronchiolitis and UTI, these studies largely required the urine culture alone (without pyuria) for the definition of UTI, which may exaggerate the UTI risk.^8^ But even if viral infection may be a risk factor for UTI, this would be an important phenomenon to describe, particularly as UTI is currently thought largely to be a nontransmissible infection.

Third, the pandemic also led to apprehension to pursue health care visits for fear of exposure to COVID-19 in the health care setting, which in turn may have led to a decrease in urine testing for UTI. For this reason, milder UTIs that would have typically been seen in the health care setting may have gone undiagnosed and untreated. Some earlier studies have reported that some UTIs resolve without treatment.^9,39,45^ In one study that analyzed 807 febrile infants younger than 3 months who did not undergo urine testing or receive antibiotic treatment on initial presentation, 61 were estimated to have a UTI. However, only 2 were ultimately diagnosed with UTI on a subsequent visit.^39^ Another study of infants evaluated for UTI who had substantial growth from catheterized urine cultures and did not receive initial antibiotic treatment reported that subsequent suprapubic aspirates yielded no growth on most infants.^9^ While the authors of that investigation attributed these findings to contamination of the initial specimen, spontaneous resolution of true infection is an alternative explanation. In addition, a randomized noninferiority trial of 253 women with UTI reported that 54% of the participants had symptom resolution at day 3 without antibiotic treatment.^45^ These studies all suggest that some cases of UTI may resolve without treatment, and even if a portion of the decrease in UTI diagnosis during the pandemic may be due to spontaneous resolution of true infections, this concept is important to highlight as we strive to avoid potentially unnecessary antibiotic treatment.

### Limitations

This study has several limitations. The database only includes commercial claims, which limits the generalizability to the US population with public insurance. We relied on diagnosis codes, which have potential for miscoding. The data set is not linked to full electronic medical records, so we did not have detailed clinical and laboratory information. Although we required evidence of an antibiotic prescription fill for our definition of UTI, misclassification still may have occurred in UTI diagnosis and similarly for measures of severity if a patient was hospitalized for an alternative diagnosis. In addition, we were unable to examine the long-term sequelae (eg, hypertension, chronic kidney disease, and kidney scarring) of UTIs that were untreated or where treatment was delayed. Future studies should evaluate whether the reduced diagnosis of UTI in this cohort translates into longer term kidney injury.

## Conclusions

In this cohort study, we report the current population incidence of UTI in children with private insurance. Using the COVID-19 pandemic as a natural experiment where the pursuit of health care services decreased and viral respiratory illnesses nearly disappeared for a short period, this study observed a decrease in UTI diagnosis during the early pandemic without statistically significant increases in the short-term measures of UTI severity. Our findings may inform discussion around the overdiagnosis and/or misdiagnosis of UTI, optimal diagnostic strategies, and definitions for pediatric UTI.

## Notes

Supplement 1. **eTable 1.** Code Handbook **eTable 2.** Most Common Antibiotics Prescribed for Children Aged 0-17 With UTI Diagnoses **eTable 3.** Measures of UTI Severity Including Hospitalization and ICU Admission, by Age and Circumcision Status **eMethods.** Statistical Model Details
eReferences

Supplement 2.
Data Sharing Statement
