# Supplementary material for: Incidence of Pediatric Urinary Tract Infections Before and During the COVID-19 Pandemic
Source: JAMA Netw Open. 2024 Jan 3;7(1):e2350061. doi: 10.1001/jamanetworkopen.2023.50061 (PMC10765266; doi:10.1001/jamanetworkopen.2023.50061)
Supplement: Supplement 1. — eTable 1. Code Handbook eTable 2. Most Common Antibiotics Prescribed for Children Aged 0-17 With UTI Diagnoses eTable 3. Measures of UTI Severity Including Hospitalization and ICU Admission, by Age and Circumcision Status eMethods. Statistical Model Details eReferences [file jamanetwopen-e2350061-s001.pdf]

Supplementary Online Content

Liang D, Wang ME, Dahlen A, et al. Incidence of pediatric urinary tract infections before and during the COVID-19 pandemic. *JAMA Netw Open*. 2024;7(1):e2350061. doi:10.1001/jamanetworkopen.2023.50061

eTable 1. Code Handbook

eTable 2. Most Common Antibiotics Prescribed for Children Aged 0-17 With UTI Diagnoses

eTable 3. Measures of UTI Severity Including Hospitalization and ICU Admission, by Age and Circumcision Status

eMethods. Statistical Model Details

eReferences

This supplementary material has been provided by the authors to give readers additional information about their work.

© 2024 Liang D et al. *JAMA Network Open.*

eTable 1. Code Handbook[a]

Definition	Codes
UTI[1,2]	[ICD-10] A36.85, N10, N11.0, N12, N13.6, N15.1, N30.*, N34.0-3, N39.0, P39.3
Dehydration	[CPT codes] 1019105, 1019330, 963.60, 963.61<br>[HCPC codes] J7042, J7120-21, S5011<br>[ICD-10 codes] P74.1, E86.0
Shock	[ICD-10 codes] R57.*, I95.*, R65.21
Sepsis, with or without shock	[ICD-10 codes] R65.21, A32.7, A40.1, A41.*, R65.20, P36.*
Acute kidney injury	[ICD-10 codes] S37*, N17.0, N17.1, N 17.2, N17.8, N17.9<br>[CPT codes] Z99.2<br>[Revenue codes] 0800, 0801, 0802, 0803
Hospitalization[3]	CPT codes 99221-99223, 99231-99233, 99218-99220, 99234-99236, 99238-99239
Hospitalization with ICU Stay[3]	CPT codes 99221-99223, 99231-99233, 99218-99220, 99234-99236, 99238-99239 AND [Revenue codes] 0200, 0201, 0202, 0203, 0204, 0206, 0207, 0208, 0209
Ambulatory setting[1,3,4]	[STDPLC code] 2 (telehealth), 11 (office), 17 (walk-in retail health clinic), 20 (urgent care), 22 (outpatient hospital), 23 (emergency room), 50 (federally qualified health center), 72 (rural health clinic)<br>[CPT code] 99441-49 with [CPT modifier] GT, GQ, 95 (telehealth)
Birth Hospitalizations[5,6]	[ICD-10 codes] O1002, O1012, O1022, O1032, O1042, O1092, O114, O1204, O1214, O1224, O134, O1404, O1414, O1424, O1494, O164, O2402, O2412, O2432, O24420-O24429, O2482, O2492, O252, O2662, O2672, O4202, O4212, O4292, O601xxx-O602xxx, O61xxxx-O82xxxx, O8802, O8812, O8822, O8832, O8882, O9802, O9812, O9822, O9832, O9842, O9852, O9862, O9872, O9882, O9892, O9902, O9912, O99214, O99214, O99284, O99314, O99324, O99334, O99344, O99354, O9942, O9952, O9962, O9972, O99814, O99824, O99834, O99844, O9A12, O9A22, O9A32, O9A42, O9A52, Z37xx-Z38xx (except Z371, Z374, Z377), 0Q820ZZ, 0Q823ZZ, 0Q824ZZ, 0Q830ZZ, 0Q833ZZ, 0Q834ZZ, 0U7C7ZZ, 0U7C8DZ, 0W8NXZZ, 10900ZA, 10900ZC, 10903ZA, 10903ZC, 10904ZA, 10904ZC, 10907ZA, 10907ZC, 10908ZA, 10908ZC, 10D00Z0-10D00Z8, 10D17Z9, 10DA7ZZ, 10D18Z9, 10DA8ZZ, 10E0XZZ, 10S07ZZ, 10S0XZZ, Z37xx-Z38xx (except Z371, Z374, Z377).<br>[CPT codes] 01960, 01961, 01962, 01963, 01967, 01968, 01969, 58611, 59300, 59400, 59409, 59410, 59414, 59510, 59514, 59515, 59525, 59610, 59612, 59614, 59618, 59620, 59622, 99360, 99460-99465, 99468-99469, 99477-99480<br>[Revenue codes] 0720-0722, 0724, 0729, 0170-0174.
Circumcisions	[CPT codes] 541.50, 541.60, 541.61
Antibiotics (Outpatient)	Therapeutic group = 02 (anti-infectives) AND therapeutic class = 4 (Aminoglycosides), 6 (Cephalosporins), 7 (Beta-lactam antibiotics), 10 (Penicillins), 12 (Miscellaneous Antibiotics), 16 (Quinolones), 17 (Sulfonamides & Combination), 19 (Urinary anti-infectives) and 20 (Miscellaneous anti-infectives).
Antibiotics (Inpatient)	[HCPC codes] J0120, J0290, J0295, J0690–98, J0710–15, J0744, J0878, J1267, J1335, J1890, J2020, J2185-86, J2510, J2540, J2543, J2700, J3000, J3260, J7682, J7685, S0039

[a]An asterisk denotes that all subcodes were included. For example, N30.* includes all codes for acute cystitis.
Abbreviations: ICD, international classification of diseases; CPT, current procedural terminology; HCPC, healthcare common procedure coding system; ICU, intensive care unit; STDPLC, standard point location.

eTable 2. Most Common Antibiotics Prescribed for Children Aged 0-17 With UTI Diagnoses

Rank	Antibiotic	%
1	Sulfamethoxazole/Trimethoprim	28.2
2	Cephalexin	17.5
3	Cefdinir	17.4
4	Nitrofurantoin	12.2
5	Amoxicillin & Combinations	11.6

© 2024 Liang D et al. *JAMA Network Open.*

eTable 3. Measures of UTI severity including hospitalization and ICU admission, by age and circumcision status.

	Hospitalization with UTI (n)	ICU Admission with UTI (n, %)	Shock (n, %)	Sepsis (n, %)	Dehydration (n, %)	Acute Kidney Injury (n, %)	LOS ≥ 4 days (n, %)
**All Children (0-17 years)**							
Overall	7,086 (2.0%)	955 (13.5%)	242 (3.4%)	1174 (16.6%)	2,176 (30.7%)	382 (5.4%)	1,697 (23.9%)
0-1 years	2,518 (12.3%)	295 (11.7%)	30 (1.2%)	411 (16.3%)	691 (27.4%)	52 (2.1%)	481 (19.1%)
2-4 years	832 (1.4%)	89 (10.7%)	16 (1.9%)	107 (12.9%)	308 (37.0%)	35 (4.2%)	178 (21.4%)
5-11 years	1,474 (1.2%)	206 (14.0%)	45 (3.1%)	213 (14.5%)	531 (36.0%)	94 (6.4%)	346 (23.5%)
12-17 years	2,262 (1.6%)	365 (16.1%)	151 (6.7%)	443 (19.6%)	646 (28.6%)	201 (8.9%)	692 (30.6%)
Female	5,466 (1.7%)	715 (13.1%)	119 (2.2%)	895 (16.4%)	1,776 (32.5%)	262 (4.8%)	1,276 (23.3%)
Male	1,620 (6.7%)	240 (14.8%)	43 (2.7%)	279 (17.2%)	400 (24.7%)	120 (7.4%)	421 (26.0%)
**Infant Subgroup (≤ 60 days)**							
Overall	NA[a]	47 (9.6%)	3 (0.6%)	82 (16.8%)	72 (14.7%)	6 (1.2%)	108 (22.1%)
Female	NA	24 (10%)	1 (0.4%)	41 (17.1%)	37 (15.4%)	2 (0.8%)	48 (20%)
Male, uncircumcised	NA	16 (10.5%)	1 (0.7%)	21 (13.7%)	22 (14.4%)	2 (1.3%)	31 (20.3%)
Male, circumcised	NA	7 (7.3%)	1 (1%)	20 (20.8%)	13 (13.5%)	2 (2.1%)	29 (30.2%)

[a]Not applicable. Hospitalization with UTI for the infant subgroup was not included in this supplemental table since it is not a measure of UTI severity for the infant population and has already been captured in Table 2.
Abbreviations: UTI, urinary tract infection; ICU, intensive care unit; LOS, length of stay

© 2024 Liang D et al. *JAMA Network Open.*

eMethods. Statistical Model Details

We used an Interrupted Time Series (ITS) model to estimate the percent change in the rate of each outcome that is attributable to the pandemic. The model is exactly analogous to the one we used in a manuscript by Schroeder et al,[3] and was defined in two steps.

In the first step, we transformed the time series to make it roughly stationary during the pre-pandemic period. This was accomplished by taking a logarithm and by applying yearly differencing, in which the seasonal effects are removed by subtracting off the value of the time series shifted by one year; the result is a new times series $y_t = \ln x_t - \ln x_{t-12}$. The datapoint representing March 2020 was discarded: lockdowns occurred in the middle of March 2020, meaning this datapoint is partially in the pre-Pandemic period and partially in the early Pandemic period. In the second step, we fit a four-parameter linear regression model; the four parameters are: 1) an intercept and 2) slope (to model the pre-pandemic trend), and two step changes for the 3) early and 4) mid pandemic periods, respectively (to model the changes during the pandemic). The final regression model structure was:

$$\ln x\_t\ (t) - \ln x(t - 1\ \text{year}) \sim \alpha + \beta_1 t + \beta_2 I_{\text{early pand.}} + \beta_3 I_{\text{mid pand.}},$$

where $I_{\text{early pand.}}$ is an indicator variable for months in the early pandemic period, and $I_{\text{mid pand.}}$ is the same for the mid pandemic period. We used autocorrelation-robust sandwich estimators for evaluate the 95% confidence intervals.[7] Models were run separately for each outcome we reported.

The output of the model is the average percent change in the rate of each outcome in each period compared to the counterfactual in which the pre-pandemic trends had continued unabated (i.e., if $\beta_2 = \beta_3 = 0.$) These percent changes are computed by exponentiating β_2 and β_3, respectively.

Full details of this statistical modeling approach, including the handling of the March 2020 datapoint, were publicly pre-registered at https://osf.io/7rh3s prior to completion of data cleaning.

© 2024 Liang D et al. *JAMA Network Open.*

eReferences

1. Afolabi TM, Goodlet KJ, Fairman KA. Association of Antibiotic Treatment Duration With Recurrence of Uncomplicated Urinary Tract Infection in Pediatric Patients. *Ann Pharmacother*. 2020;54(8):757-766. doi:10.1177/1060028019900650

2. Germanos G, Light P, Zoorob R, et al. Validating Use of Electronic Health Data to Identify Patients with Urinary Tract Infections in Outpatient Settings. *Antibiotics*. 2020;9(9):536. doi:10.3390/antibiotics9090536

3. Schroeder AR, Dahlen A, Purington N, et al. Healthcare utilization in children across the care continuum during the COVID-19 pandemic. *PLOS ONE*. 2022;17(10):e0276461. doi:10.1371/journal.pone.0276461

4. Tiwari T, Marinucci J, Tranby EP, Frantsve-Hawley J. The Effect of Well Child Visit Location on Preventative Dental Visit. *Children*. 2021;8(3):191. doi:10.3390/children8030191

5. Sarayani A, Wang X, Thai TN, Albogami Y, Jeon N, Winterstein AG. Impact of the Transition from ICD–9–CM to ICD–10–CM on the Identification of Pregnancy Episodes in US Health Insurance Claims Data. *Clin Epidemiol*. 2020;12:1129-1138. doi:10.2147/CLEP.S269400

6. Chua KP, Fendrick AM, Conti RM, Moniz MH. Out-of-Pocket Spending for Deliveries and Newborn Hospitalizations Among the Privately Insured. *Pediatrics*. 2021;148(1):e2021050552. doi:10.1542/peds.2021-050552

7. Kewey WK, West KD. A simple, positive semi-definite, heteroskedasticity and autocorrelation consistent covariance matrix. Published online April 1986.

© 2024 Liang D et al. *JAMA Network Open.*
