# Supplementary material for: Incidence of Pediatric Urinary Tract Infections Before and During the COVID-19 Pandemic
Source: JAMA Netw Open. 2024 Jan 3;7(1):e2350061. doi: 10.1001/jamanetworkopen.2023.50061 (PMC10765266; doi:10.1001/jamanetworkopen.2023.50061)
Supplement: Supplement 2. — Data Sharing Statement [file jamanetwopen-e2350061-s002.pdf]

Data Sharing Statement

Liang. Incidence of Pediatric Urinary Tract Infections Before and During the COVID-19 Pandemic. *JAMA Netw Open*. Published January 03, 2024. doi:10.1001/jamanetworkopen.2023.50061

Data
Data available: No
